# Mammary carcinoma behavior is programmed in the precancer stem cell

**DOI:** 10.1186/bcr2104

**Published:** 2008-06-03

**Authors:** Patrizia Damonte, J Graeme Hodgson, Jane Qian Chen, Lawrence JT Young, Robert D Cardiff, Alexander D Borowsky

**Affiliations:** 1 Center for Comparative Medicine, Department of Pathology and Laboratory Medicine, UC Davis, County Road 98 and Hutchison Drive, Davis, California 95616, USA; 2 UCSF Department of Neurological Surgery, 1855 Folsom Street, Suite 535 Box 1631 San Francisco, California 94143-1631, USA

## Abstract

**Introduction:**

The 'MINO' (mammary intraepithelial neoplasia outgrowth) mouse model of ductal carcinoma *in situ *(DCIS) consists of six lines with distinct morphologic phenotypes and behavior, each meeting experimentally defined criteria for 'precancer'. Specifically, these lines grow orthotopically in cleared mammary fat pads and consistently progress to an invasive phenotype that is capable of ectopic growth. Transition to carcinoma has a consistent latency for each line, and three of the lines also exhibit pulmonary metastatic potential.

**Methods:**

Gland cleared orthotopic transplanted precancer MINO tissues were analyzed by bacterial artifical chromosome and oligo array comparative genomic hybridization, microsatellite PCR, and telomerase repeat amplification assay. MINO cells were dissociated and cultured in three dimensional culture and transplanted in syngeneic gland cleared mammary fat pads.

**Results:**

Comparative genomic hybridization shows that the precancer and invasive tumors are genetically stable, with low level changes including whole chromosome gains in some lines. No changes are associated with progression, although spontaneous focal amplifications and deletions were detected occasionally. Microsatellite analysis shows a low frequency of alterations that are predominantly permanent within a MINO line. Telomerase activity is increased in both the MINO and the derived tumors when compared with normal mouse mammary gland. Dissociation of the precancer lesion cells and three dimensional 'spheroid' culture of single cells reveals a bipotential for myoepithelial and luminal differentiation and the formation of unique three-dimensional 'MINOspheres'. These MINOspheres exhibit features that are intermediate between spheroids that are derived from normal and carcinoma cells. Transplantation of a single cell derived MINOsphere recapitulates the outgrowth of the precancer morphology and progression to carcinoma.

**Conclusion:**

These data establish a precancer 'stem' cell that is capable of self-renewal and multilineage differentiation as the origin of invasive cancer. Within the context of this model, these cells have programmed potential for latency and metastasis that does not appear to require sequential genetic 'hits' for transformation.

## Introduction

Ductal carcinoma *in situ *(DCIS) refers to phenotypically heterogenous lesions that are defined by a common property – increased risk for cancer at the site of the biopsy [1-3]. This property implies a direct clonal progression from DCIS to invasive carcinoma, and it is the conceptual basis for current DCIS treatment [4]. DCIS can be subtyped and graded with implications for latency to invasion and the likelihood of spread/recurrence [5]. Although controversial, DCIS does not appear to progress from lower grades or low risk types to higher grades or higher risk types *en route *to cancer or upon recurrence, which suggests a relatively stable population. In summary, the clinico-epidemiologic pathology data support the hypothesis that the cells of DCIS may have a programmed potential for phenotype, possibly including progression to invasion, metastasis, hormone receptor expression, and therapeutic resistance.

We have used the combination of mammary transplantation [6] with derivative genetically engineered mouse mammary gland to create a mouse model of DCIS that recapitulates the clinico-epidemiologic observations in human disease [7]. The models are referred to as mouse *mammary intraepithelial neoplasia outgrowths *(MINOs). The biologic behavior of these tissues is operationally defined by the 'test-by-transplantation', in which each of the six MINOs meets the following transplantation criteria: grows in gland-cleared fat pad (orthotopic); does not grow in the subcutis (ectopic); does not senesce over many generations of transplantation; and consistently transforms to a phenotype characterized by an ability to grow in the subcutis (ectopic). Of particular interest and relevance to our understanding of human breast cancer progression are three preliminary findings. First, three of the lines metastasize and three do not. This finding is consistent over subsequent generations of MINO transplantation [8]. Second, the time or latency to transformation is consistent within a given MINO line over multiple transplant generations, although different lines have different latencies. Third, gene expression analysis and hierarchical clustering show that a MINO and the transformed lesion arising within it are more closely related than any two MINO lines or any two transformed tumors [8,9]. Because the recipient mice are genetically identical but immune-intact FVB mice, and because the origins of the MINO lines are two genetically identical transgenic mice, Tg(MMTV-*PyVmT*) on an FVB background, this can be considered a model of human DCIS without variation in genetic susceptibility loci.

These characteristics of the MINO model support the hypothesis of a preprogrammed behavior at the precancer stage. In this report we show that these potentials are pre-encoded in individual cells within the complex MINO tissue. This individual cell precancer reinitiating potential is supported in part by evidence that the precancers and resulting cancers are clonally derived and telomere stabilized. However, the truest definition of initiating cell behavior in single precancer cells employs a functional analysis *in vivo*. For this MINO model, previously published data [10] and Additional files presented here show genetic clonality and genomic stability by medium-resolution and high-resolution array comparative genomic hybridization (CGH). Despite the fact that a large number of cell types are co-transplanted in each generation, the precancer cells and the tumor cells that arise within the precancer share this apparent clonal origin.

In this report we also show by telomerase analysis that both the precancer and invasive carcinomas are characterized by increased activity, which is sconsistent with the genomic stability observed in these tissues. We show that rare individual cells within the MINO tissue are responsible for this clonal but phenotypically heterogeneous biology. Finally, we show that the criteria of cancer stem cells (as defined by the American Association for Cancer Research task force on cancer stem cells [11]) for self-renewal and the ability to reconstitute the neoplasm are present in these individual cells. This finding, in combination with the previously published findings of conserved latency to invasive carcinoma and conserved metastatic potential, suggests that the cancer phenotype is programmed or imprinted in the precancer initiating cell [11].

## Materials and methods

### Microsatellite PCR

Microsatellite PCR was performed using the NaOH extracted DNA samples (normal mammary gland, MINO, and cancer tissue) above in multiplexed assays to interrogate loci spread over the mouse genome. (The panel was originally developed for mouse strain background analysis.) The primers used were all from the collection developed at the Massachusetts Institute of Technology. The following cycling conditions of PCR were used: 23 cycles of 95°C for 10 minutes, 85°C for 10 minutes, and 95°C for 1 minute; 23 cycles of 55°C for 0.5 minutes; 23 cycles of 72°C for 0.75 minutes; and 72°C for 30 minutes and then 5°C. PCR products were replicated with 10:2:1 formamide:dye:genscan ladder and then denatured for 3 minutes at 95°C and neutralized for 1 minute at 4°C. The samples were run on 7% denaturing polyacrylamide gel. Sample results are shown as nucleotide length performed via ABI sequencing column analysis (Applied Biosystems, Foster City, CA, USA). Analysis was performed using STRand software created by the Veterinary Genetics Laboratory at the University of California, Davis.

### Telomerase activity detection

Telomerase activity was evaluated using the TRAPeze Telomerase Detection Kit (Chemicon International, Temecula, CA, USA) using 200 μl of 1×CHAPS lysis buffer/100 mg of MINO tissue or MINO tumor. Total protein extracts were quantified using the Bradford assay and diluted in collection buffer (1×CHAPS) to concentrations of 50 μg/ml, 10 μg/ml, and 2 μg/ml for incubation with the telomere repeat DNA template. The PCR program used was as follows: 30 to 33 cycles of 94°C for 30 seconds, 59°C for 30 second, and 72°C for 1 minute. We ran 25 μl of this PCR product on a 10% to 12% nondenaturing PAGE gel in 0.5× TBE buffer, for 1.5 hours at 200 V. Thirty-six base pairs (bp) was used as internal control for PCR. The gel was stained with ethidium bromide 1:10,000 for 30 minutes and then destained for 30 minutes. The ratio of band intensity (50 bp:36 bp) was used for quantification, and the average for this ratio divided by the protein concentration recorded for each sample reaction.

### Single cell isolation and spheroid analyses

Normal, MINO, and tumor tissues were obtained from wild-type FVB females at 4 weeks after transplantation for MINO tissue or when tumors arose. After mechanical mincing with a McIlwain tissue chopper (Mickle Laboratory Engineering, Guildford, UK), the tissues were placed in serum-free digestion medium (F_12_/Dulbecco's modified Eagle's medium [DMEM] 1:1 with 10 mmol/l Hepes [Invitrogen, Carlsbad, CA, USA], 2× P+S, 2% bovine serum albumin fraction V [Invitrogen], 5 mg/ml Insulin [Sigma, St Louis, MO, USA], 0.5 mg/ml hydrocortisone [Sigma], 10 ng/ml cholera toxin [Sigma], and 3 mg/ml Collagenase [Worthington Biochemical Corp., Lakewood, NJ, USA] and digested on a stirrer plate for 16 hours at room temperature. The resulting organoid suspension was pelleted at 80 *g *for 4 minutes and sequentially washed with F_12_/DMEM and pelleted at 1,000 rpm for 4 minutes. A suspension of single cells was obtained by sequential dissociation of the fragments by gentle pipetting and incubation at 37°C for 1 to 2 minutes in 0.25% trypsin. After adding 0.1 mg/ml DNase I, the sample was incubated for a further 5 minutes at 37°C and then an equal volume DMEM with 10% fetal calf serum was added to stop the trypsinization. Remaining clumps were removed by filtration through a 40 mm cell strainer. For three-dimensional culture the single cells were pelleted and resuspended using cooled pipettes in cold BD Matrigel matrix [BD Biosciences, Bedford, MA, USA] and plated in 24-well plates. The plates were incubated at 37 C for 30 minutes and then the growth medium was added (mammary epithelial growth medium with 1× B27, 20 ng/ml epidermal growth factor [BD Biosciences], 20 ng/ml basic fibroblast growth factor [BD Biosciences], heparin, and 20 mg/ml insulin [Sigma]).

After 2 weeks in Matrigel the spheroids arising from single cells were transplanted or fixed in 10% formalin, embedded first in HistoGel Biopsy Gel [Richard-Allan Scientific, Kalamazoo, MI, USA] and then in paraffin. Transplantations of spheroids were done with the aid of a Zeiss Stemi 2000-C dissecting microscope (Carl Zeiss Inc., Thornwood, NY, USA). Spheroids, cultured within Matrigel, were identified and located with the dissecting microscope. A Dumont #5–45 forceps [Dumoxel-Fine Science Tools Inc, Foster City, CA, USA] was used to gently hold and lift individual spheroids from the culture dish. Each MINOsphere(s) was transplanted into the cleared inguinal fat pad of FVB/N female mice.

### Histology and immunohistochemistry

Four micrometer thick paraffin sections were stained with Mayer's hemotoxylin and eosin or immunostained as described previously [8]. The following primary antibodies were used with the VECTASTAIN ABC Elite Kit (Vector Laboratories, Burlingame, CA, USA): guinea pig anti-cytokeratin (CK) 8–18 (1:1,000; RDI- Research Diagnostics Inc, Concord, MA, USA), sheep anti-CK14 (1:400; Binding Site Inc., San Diego, CA, USA), rabbit anti-Ki67 (1:800 Neomarker, Fremont, CA, USA), and rabbit anti-CK5 (1:1,000 Abcam Inc., Cambridge, MA, USA). Dako Ark kit (Dako, Carpinteria, CA, USA) was used for immunohistochemistry with mouse anti-smooth muscle actin (SMA; 1:1,000; Sigma) antibody. Images of slides were captured using 20× and 40× objectives on a AxioScope microscope (Carl Zeiss Inc.) with AxioCam camera and processed using Adobe Photoshop 7.0 (Adobe Systems, Inc., San Jose, CA, USA) software.

## Results

### MINO growth properties

**Figure 1:**
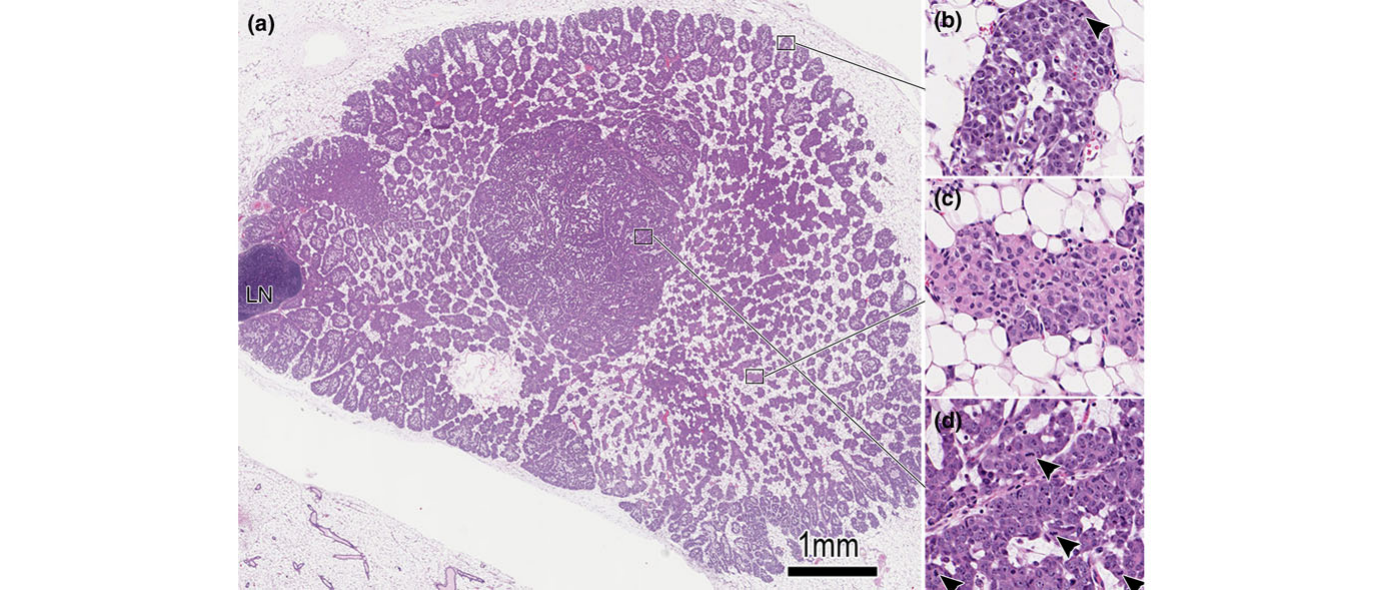
Histomorphology of the MINO model mammary precancer with early invasive carcinoma. **(a) **Low-power hematoxylin and eosin histomorphology of the MINO precancer filling the precleared fat pad. Intact gland is seen for comparison in the bottom left corner. The lymph node is seen (LN) at the left, and no invasion of the node by precancer tissue is seen. **(b) **The edge of the precancer is seen at higher power and is similar to a normal developing mammary gland terminal end bud with mitoses (arrowhead) and remodeling via apoptosis to clear the luminal space. In the area of precancer in the center of the growth there is an organized relationship between the transplanted MINO tissue and the host stroma. **(c) **The MINO cells differentiate to form acinar and ductal structures with high intralesional cell heterogeneity. **(d) **In this heterogeneous differentiated zone of precancer tissue, an area of transformation to invasive carcinoma is characterized by increased mitoses (arrowheads) and much less residual host stromal tissue. MINO, mammary intraepithelial neoplasia outgrowth.

Each of the six individually derived MINO transplant lines maintains consistent genomic changes through serial generations and between recipients within a line and within a transplant generation. The MINO lines have been maintained in serial transplantation for between 50 and 60 generations (depending on the line) without senescence. Considerable phenotypic heterogeneity exists between lines and within a line. Several important behaviors have remained consistent, however, and these define these mammary outgrowths as 'mammary intraepithelial neoplasia' (MIN) or precancer. Specifically, the MINO tissues grew only in the cleared fat pad and were contact inhibited by the edge of the pad or by an adjacent mammary tree. As shown in Figure 1, the MINOs grow to fill the cleared fat pad with a leading edge mimicking normal gland development, including 'terminal end bud' extensions into the surrounding fat pad (Figure 1b). Just as in the developing normal gland, this proliferative and expanding area is characterized by proliferation followed by apoptosis and differentiation leading to a 'differentiation zone' (Figure 1c) in the MINO. Within this differentiation zone of MINO tissue, invasive carcinomas arise (Figure 1d). These are characterized by more spherical growth, which is not limited to the dimensions of the fat pad, and by the potential for ectopic growth if transplanted subcutaneously.

### Microsatellite analysis of MINO and tumor

Cancers may harbor genetic instability undetected by karyotype or CGH analyses, although human breast cancer is usually characterized by aneuploidy, abnormal karyotypes, and abnormal genomic content by CGH. Nevertheless, instability in some cancers may be comprised of single base mutations or amplifications or deletions that are too small to be detected by CGH. A sensitive assay to detect instability unresolved by CGH is the analysis of microsatellite repeats within the genome. Dinucleotide repeats are the most sensitive, followed by trinucleotide and then tetranucleotide repeats. Repeat length of microsatellites distributed throughout the mouse genome was determined in multiplexed PCR assays.

The results are summarized in Table 1 and Additional file 1 alongside the CGH results, and complete results are presented in Additional file 2. Each red filled box in Additional file 1 represents a repeat length different from the normal control. The number of microsatellite changes is small. Additional file 2 and the data summary presented in Table 1 and in the Additional file 1 shows line A with only four changes of 192 repeats tested, with two in common between MINO and tumor and two unique to the tumor. Line D shows six changes of the 192 tested in the MINO, with five of these and one additional in the paired tumor (five additional changes were uninformative because of a failed reaction for one of the samples). Line 11 shows seven changes in the MINO and six of them with one additional in the tumor. Line B shows five changes with one additional in the tumor. Line 4 shows seven changes in the MINO with six of the seven present in the tumor.

**Table 1:** Summary of CGH BAC array and multiplexed microsatellite PCR length changes in the MINO precancer tissue and paired invasive carcinoma

Line designation/G0 generation #		Generation G0		Generation G0+1	
		
		MINO	Paired tumor	MINO	Paired tumor
Line A	CGH whole chromosome	+ch13WC	Same	→ Same	→ Same +ch11WC
G0 = 12th	CGH locus changes	+ch5BAC1	Same	→ Same +ch5BAC2	→ Same
	MS			d5–95; d7–253	→ Same +d6–123; d9–90
(Replicate animal)	CGH whole chromosome			Same	Same +ch11WC
	CGH locus changes			Normal	Normal
Line D	CGH	Normal	Normal		
G0 = 12th	MS	d1–24; d2–149; d4–178; d12–109; d12–182; d14–170	→ Same less d1–24; plus d7–165		
Line 11	CGH whole chromosome	Normal	Normal		
G0 = 11th	CGH locus changes	Normal	+ch5distalG1:G3 (17 BACs); +ch11BACs3–5		
	MS	d2–149; d4–166; d5–113; d8–205; d11–61; d16–131; d18–19	→ Same less d2–149; plus d9–182		
Line B	CGH	+ch1WC, +ch2WC, +10WC, +11WC,	→ Same less +ch11WC, plus +ch15WC		
G0 = 14th		+ch3BAC6, +ch17BAC7	→ Same plus +ch5BAC1, +ch5BAC2, del ch7BAC8, del ch8BAC9, del ch10BAC10		
	MS	d6–138, d8–93, d8–120, d10–14, d11–61	→ Same +d17–93		
Line 4	CGH whole chromosomes	+ch1WC	+ch2WC	+ch2WC	→ Same
G0 = 13th	CGH locus changes	+ch5BAC1, +ch6BAC11	→ Same less ch6BAC11	Normal	del-ch2BAC12, del-ch6BACBAC13, del-ch18BAC14, del-ch18BAC15
	MS			d1–102, d2–149, d12–109, d12–182, d12-nds, d15–175, d3–57	→ Same less d2–149
Line 6	CGH whole chromosome	Normal	Normal	+ch1WC	→ Same
G0 = 11th	CGH locus changes	Normal	Normal	+distal ch5G1:G3 (18 BACs), +ch3BAC2	→ Same
(Replicate animal)	CGH whole chromosome	Normal	Normal		
	CGH locus changes	Normal	Normal		

The initial serial transplant generation (G0) tested is given for each line, and, for several lines, a successive generation (G0+1) was tested. Replicate animals transplanted at the same time from the same donor (previous generation) were also tested. The changes from normal are listed as comparative genomic hybridization (CGH) detected whole chromosome (WC) gains (+ch#), CGH focal locus amplifications (+) and deletions (del). The bacterial artificial chromosome (BAC) clones are numbered as follows: BAC1 = RP23 422O10; BAC2 = RP23 93M18; BAC3 = RP23 276J22; BAC4 = RP23 295O13; BAC5 = P1–7858; BAC6 = RP23 233B21; BAC7 = RP23 193I17; BAC8 = RP23 413L18; BAC9 = RP23 23D17; BAC10 = RP23 421E11; BAC11 = RP23 127M17; BAC12 = RP23 129L11; BAC13 = RP23 393H9; BAC14 = RP23 27B11; and BAC15 = RP23 35M16. Most of the BACs are from Roswell Park (RP) 23 collection. Also shown are microsatellite (MS) changes compared with normal control (MSs were not tested for line 6). Specifics of MS changes are available in Additional file 2. The arrows indicate comparisons with samples either from a previous generation or from the MINO preceding the invasive cancer (**→**) or from a different transplant in the same generation listed in rows above (). MINO, mammary intraepithelial neoplasia outgrowth.

By criteria for genetic instability phenotypes in human cancers, these results would be considered genomically 'stable' [12]. Most changes detected were seen in common between the MINO and the associated invasive carcinoma. For a few loci, the MINO and tumor did have different changes, but none of these unique tumor changes were shared between lines. Changes seen in the tumor and not the MINO might represent a very low frequency of genetic instability. Changes seen in MINOs and not tumors imply divergent evolution of the MINO clone, with the tumor arising from an area in the MINO that did not harbor the specific change. The majority of changes were unique to the MINO line, and still present in the invasive tumor in the line, implying that the invasive tumor arises from cells of the precancer MINO.

### Telomerase activity/telomerase repeat amplification protocol assay

**Figure 2:**
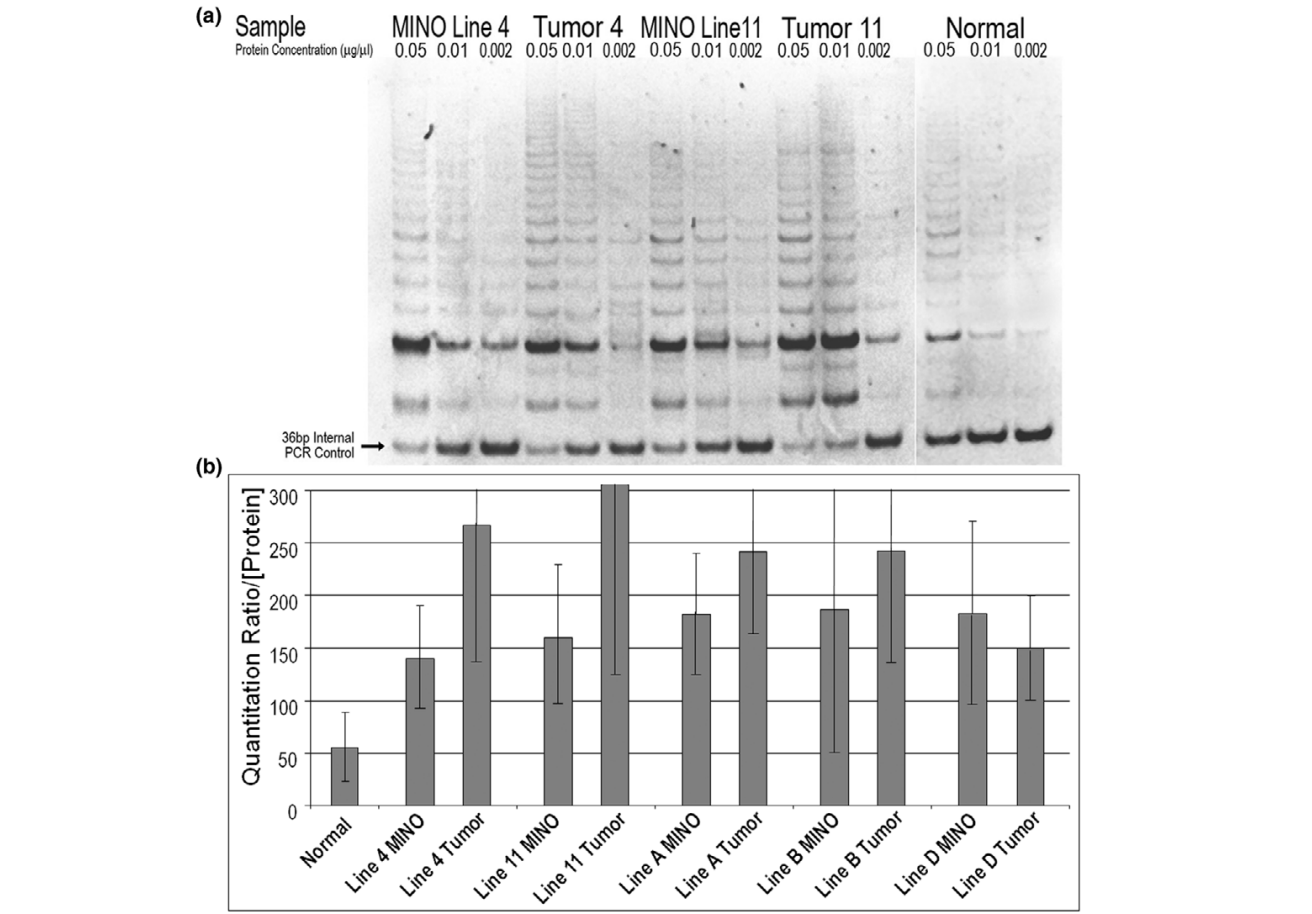
Telomerase activity of MINO and tumor tissues. Telomerase activity by telomerase repeat amplification protocol (TRAP) assay. Representative ethidium stained polyacrylamide gel electrophoresis of the PCR products resulting from protein extract incubation with artificial telomere repeat template showing a bright 50 bp band with 6 bp increasing length ladder. At the bottom of the gel is a 36 bp internal PCR control, with PCR optimized to be semi-competitive with the extended telomere repeats produced by the telomerase protein derived from each sample. The protein samples are diluted in buffer and then added to the reaction mixture to provide a final reaction mixture concentration as listed (top of each lane). Matched MINO and tumor samples were tested. **(a) **Lines 4w4 and 4w11 are shown, with normal mouse mammary epithelium control (separated from the stroma by partial tissue dissociation and centrifugation) shown in the right three lanes. **(b) **Quantitation by comparing the band intensity with the internal control for multiple samples is shown, with standard deviation of the mean depicted for each bar. Line 11 tumor had the highest levels, at a mean of 500 (not shown), and all samples except for line B MINO were statistically significantly different from the normal control. There was a high level of variability between assay runs, depicted by the size of the error bars, but the qualitative data were clear, as shown in the gel (panel a). bp, base pairs; MINO, mammary intraepithelial neoplasia outgrowth; TRAP, telomerase repeat amplification protocol.

In order to evaluate the activity of telomerase in the MINO tissues and matched tumors as compared with normal mammary epithelium, we performed the telomerase repeat amplification protocol (TRAP) assay. A sample of the polyacrylamide gel electrophoresis is shown in Figure 2a. Here the TRAP assay showed the formation of an extended ladder of six base repeats but with a quantitative competition between the telomerase repeat template and the internal control (36 bp) for PCR. The ratio of band intensity (50 bp:36 bp) was used for quantification. The average for this ratio divided by the protein concentration was recorded for each sample. The resulting representation of quantitative data is shown in Figure 2b. This histogram shows a consistently higher telomerase activity in MINO and tumor tissues compared with normal tissue. Significant variability between samples was observed. Some lanes were considered 'failed reactions' and were discarded. The variability contributes to the size of the error bars, but in all cases, except for the line B MINO, the error does not overlap with normal. The data also present a higher average telomerase activity in tumors relative to the MINOs in which they arose, but this is not statistically significant. This trend may be related to an increased contribution from normal stroma in the MINO tissue compared with the tumor tissue samples. In many studies the subjective analysis of ladder intensity and height (repeat length) was more clearly increased from normal than indicated by the quantitative ratio, but this subjective analysis (as illustrated in Figure 2a) was not quantified. Notably, consistent with the literature, the normal mouse mammary gland has significantly higher telomerase activity than normal human tissues.

### Cell dissociations and organoid culture

Because the serial transplant tissues are composed of multiple cell types, the precancer outgrowths are not precisely a clonal derivative of a single cell. In order to overcome this objection, we initially attempted to grow the precancer cells in adherent cell culture using the techniques previously described for mouse mammary carcinoma cell line derivations [13]. This resulted in either poorly growing cultures or cultures of cells that, after serial passage, acquired characteristics of invasive carcinomas/tumors. Specifically, they acquired the ability to grow in ectopic locations (subcutaneous). In contrast, cell dissociations of MINO tissues and injection of these cells or mammospheres grown in suspensions from these cells into the precleared fat pad resulted in MINO phenotypes, which did not grow in subcutaneous/ectopic sites. Thus, conversion of the precancer to invasive carcinoma was not related to cell dissociation alone.

Therefore, three-dimensional culture techniques were adapted and optimized. Several initial considerations were critical to the experiments. First, MINO tissues for cell dissociations and culture were harvested early enough to ensure that no nascent tumors were present in the precancers, using *in situ *microscopic observation and latency data derived from both time course study and *in vivo *imaging [8,14]. Although the latency time varies for each line, MINO tissues were generally harvested before 5 weeks for all dissociation studies. Second, after serial cell dilutions, the cells were plated in an artificial extracellular matrix (Matrigel) to prevent individual cells from contacting or 'bumping into' each other, sticking, and cooperating in their subsequent growth. This permitted assessment of the potential for growth and morphology of individual cells. Because of the importance of the potential of each individual cell, the rate of doublet and groups of cells persisting through the dissociations was assessed. In addition, doublet and groups of cells in the matrix-immobilized cultures were marked and followed over time to compare their fate with that of the predominant single cells. These two approaches allowed assessment of the growth properties of single isolated cells.

Three-dimensional cultures of MINO derived by cell dissociation were performed in parallel with dissociated cells from normal (either virgin or pregnant) mouse mammary gland tissue and with invasive mammary tumor tissue.

**Figure 3:**
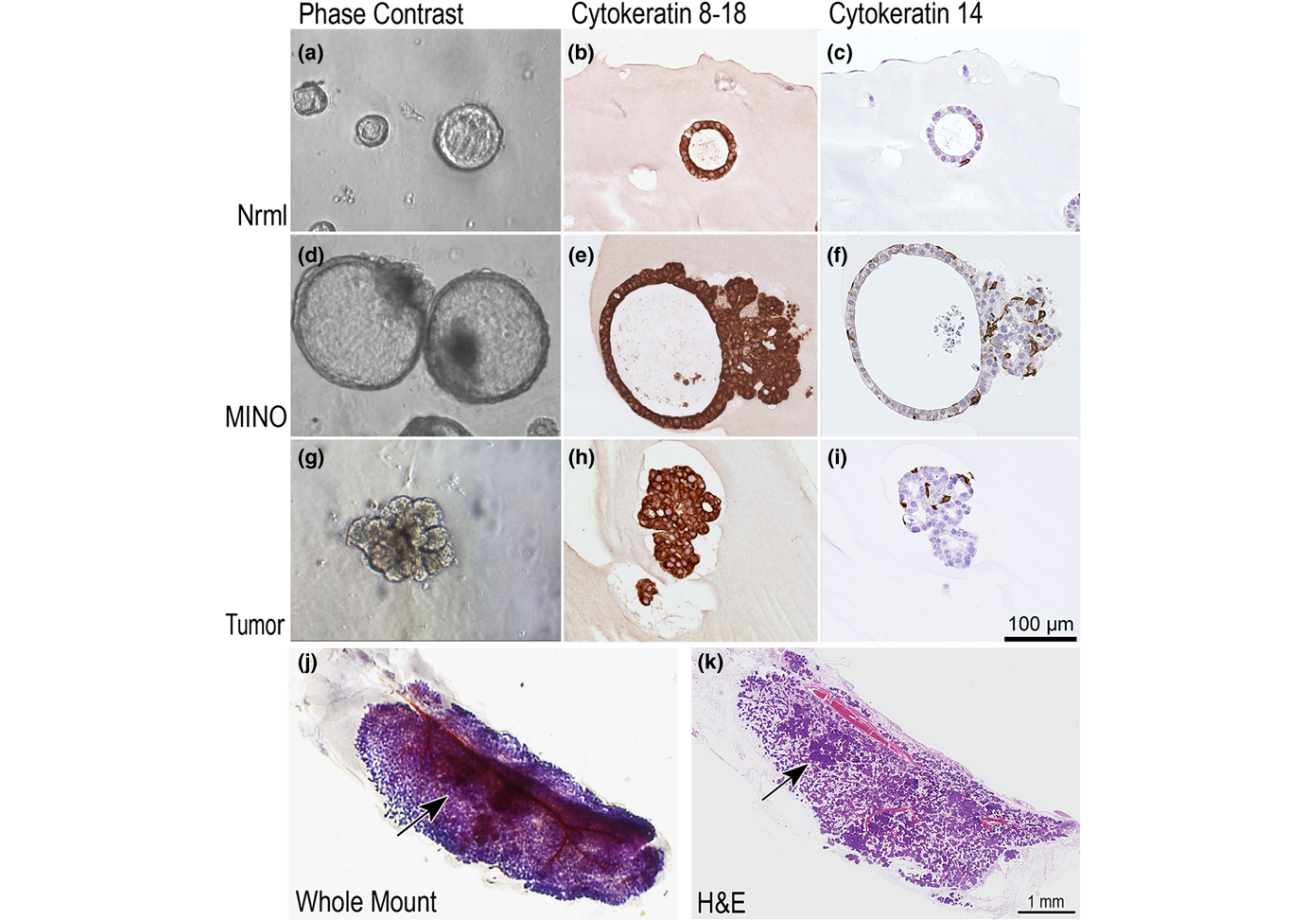
Three-dimensional culture of single cells from normal prelactating mammary gland, MINO precancer, and invasive carcinoma. **(a, d, g) **Inverted microscope phase contrast images as well as histologic images generated from paraffin-embedded 4 μm sections of the three-dimensional cultures stained by immunohistochemistry with **(b, e, h) **CK8–18 or **(c, f, i) **CK14 are shown. The magnification scale (lower right panel i) is identical for all histology panels and approximate for the inverted microscopy photographs. CK8–18 confirms that the major cell population is a luminal phenotype, but CK14 shows that there is also myoepithelial differentiation of single cells, documenting bipotential of the individual cells giving rise to these three-dimensional structures. A single MINOsphere from line 4w4 was transplanted from the three dimensional culture into the gland cleared fat pad of a 3-week-old female FVB/n mouse. The mammary gland was removed 10 weeks after transplant and a whole mount mammary gland preparation was made with hematoxylin stain to visualize cell density **(j)**. After photography, the same gland was processed for histologic sectioning, and the resulting 4 μm hematoxylin and eosin stained tissue section is shown **(k)**. At least three foci of tumor are seen in the differentiation zone of the MINO. One is indicated by the arrow. CK, cytokeratin; MINO, mammary intraepithelial neoplasia outgrowth.

A minority of single cells in each group gave rise to three-dimensional growths in culture. In the normal mammary gland controls, about 1% to 9% of cells gave rise to small spheroids that grew in size gradually but retained a spherical shape (spheroids), with a hollow center appearing after the sphere reached a sufficient size for identification (Figure 3a). The morphology of the spheroids and the percentage of cells that are capable of this growth were unchanged between virgin (multiple glands used from multiple mice) and prelactating normal mammary gland cells. Histologic sections of these normal spheroids revealed a clear double layer of cells with uniform, larger luminal mammary epithelial cells lining the center of a hollow cavity, surrounded by a second layer of flatter cells with smaller nuclei forming a discontinuous web.

Immunohistochemistry confirmed the cell types with CK8–18 marking the inner luminal cells and CK14 marking the outer basal cells (Figure 3b–c).

Approximately 1 in 10 isolated single tumor cells gave rise to a variety of morphologic shapes in the three-dimensional culture. Irregular and roughly spherical shapes were observed, but organized central cavities were not (Figure 3g). In the histologic sections the tumors formed irregular aggregates, with small groups of cells sometimes forming gland-like lumina. Immunohistochemistry showed both luminal CK8–18 positive and basal CK14 positive cell types in the tumor organoids (Figure 3h–i).

In contrast, single MINO isolated cells in three-dimensional cultures formed three-dimensional growths from roughly 0.08% ± 0.02% of cells. These 'MINOspheres' grew faster than the normal cell spheroids and had a similar appearance, with a prominent cavity forming in the center of a spherical growth (Figure 3d). However, the MINOspheres were characterized by a polarized aggregate of more irregular cell growth, which was not seen in the spheroids from normal mammary gland. Histologic assessment of the 'MINOspheres' revealed an intermediate phenotype between normal and tumor. They had organized two-cell layer spheres with larger lumina that account for the greater part of the volume of the growth. Furthermore, one pole of the spheroid contained an irregular tumor-like growth, which was continuous with the edge of the spheroid. Immunohistochemistry confirmed that the sphere was comprised of inner luminal CK18-positive epithelial cells, surrounded by a more discontinuous outer layer of CK14-positive myoepithelial cells (Figure 3e–f). The irregular pole was composed of a more disorganized mixture of luminal epithelial and myoepithelial cells. Additional immunohistochemistry (Ki67) demonstrated increased proliferation in the disorganized region (data not shown).

The MINOsphere phenotype was consistent through multiple iterations of the experiment and was consistent between different lines of MINO. In particular, lines 4, D, and B were studied over three independent serial generations. All showed the MINOsphere phenotype intermediate between normal and tumor cells. Some differences were observed between MINOspheres from different lines. The clearest example of phenotypic differences was exhibited by MINO line B. Line B single cells formed MINOspheres with the area of disorganized cells, but the spheres became filled with inspissated proteinaceous debris (not shown).

### Transplantation of single cell derived MINOspheres

The MINOspheres resulting from three dimensional cultures in 24-well plates were transplanted in precleared fat pads of syngeneic mice. Often, only one MINOsphere per well was observed in wells plated with 10,000, 5,000, or 1,000 cells. Under direct observation with a Zeiss dissecting microscope, the single MINOsphere could be removed from the well and transplanted into the precleared fat pads of syngeneic 3-week-old female FVB/n mice. As a control, a similar area of matrigel but from a well without any apparent MINOspheres was separately transplanted. Fifty-five percent (*n *= 20) of MINOsphere transplants resulted in MINO growth in the precleared fat pad, whereas no growth (0%, *n *= 4) was seen with transplants of the controls. A two-sided χ^2 ^test of these data results in *P *= 0.046. The MINOsphere derived outgrowth morphologically matched the original MINO tissue outgrowth (Figures 1 and 3k).

Importantly, the MINOsphere transplants retained the behavioral properties of terminal end bud-like growth to fill the fat pad, followed by heterogeneous differentiation and focal transformation into invasive carcinoma (Figure 3j, arrow). The latency time to the invasive carcinoma was similar to the previously reported latencies for each line. Metastatic potential has not been retested.

### Comparative pathology of MINOsphere derived and donor serial tissue transplant outgrowths

**Figure 4:**
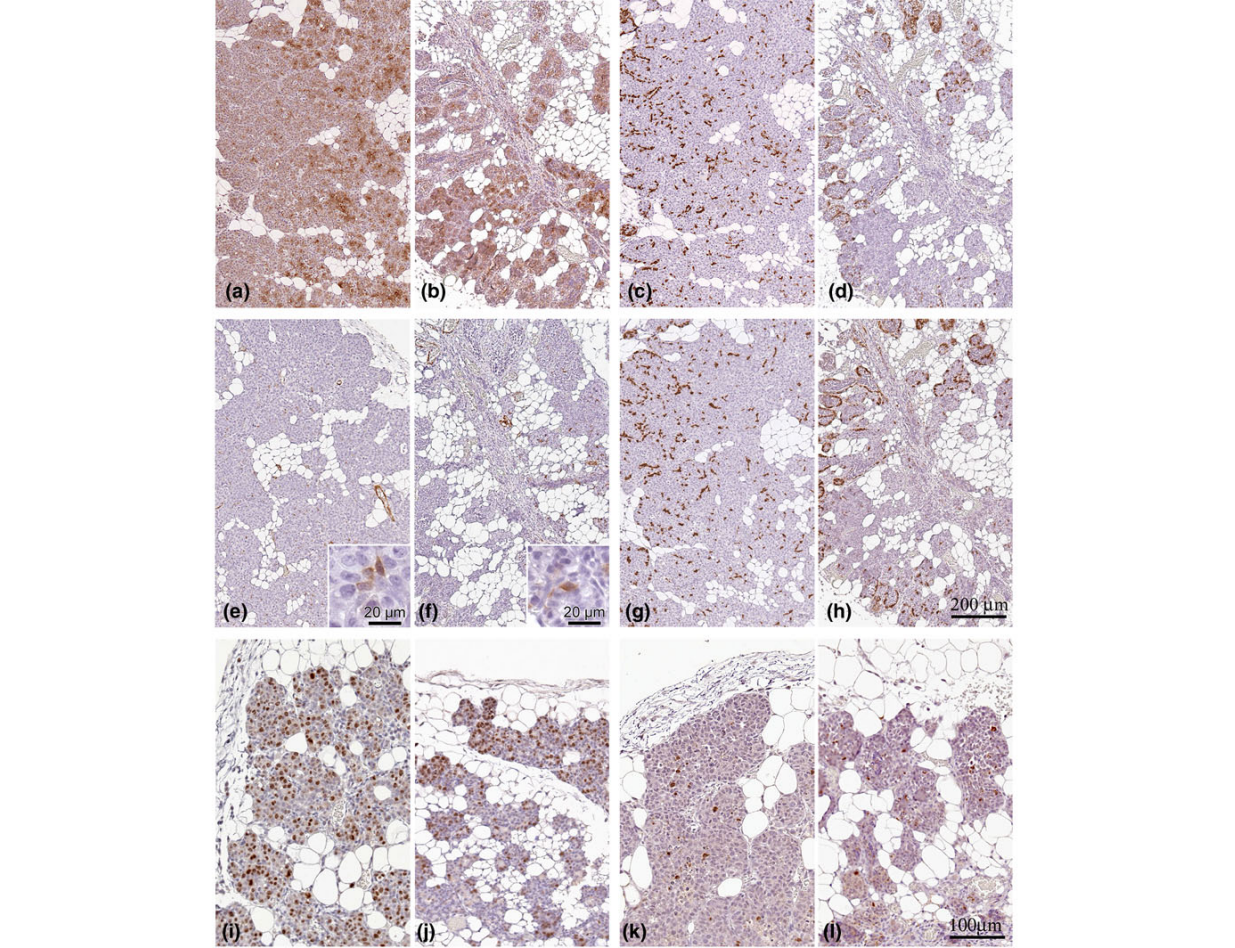
Immunohistochemistry profile of MINOsphere-derived outgrowth versus the original MINO tissue outgrowth. The outgrowth derived from a MINOsphere transplant **(a, c, e, g, i, k) **shows analogous expression compared with the MINO tissue outgrowth from the donor **(b, d, f, h, j, m) **for the following: luminal epithelial, CK8–18 (panels a and b); basal epithelial/myoepithelial, CK14 (panels c and d) and CK5 (panels g and h); myoepithelial/smooth muscle, SMA (panels e and f insets show higher magnification of SMA-positive cells within the epithelial layers); and proliferation/apoptosis, Ki67 (panels I and j)/caspase3 (panels k and l). Panels a to h are of identical magnification, with the 200 μm scale bar shown (panel h). Panels i to l are identical magnification with the 100 μm scale bar shown (panel l). CK, cytokeratin; MINO, mammary intraepithelial neoplasia outgrowth; SMA, smooth muscle actin.

The phenotypes of MINOsphere derived outhgrowths matched the original (donor serial transplant) outgrowths in comparative pathology analysis. This comparison was also validated by immunohistochemistry. The immunohistochemistry analyses confirm the same patterns and distributions of expression of CK8–18, CK5, CK14, and SMA. Ki67 and activated caspase 3 immunohistochemistry also showed matching proliferation and apoptotic rates (Figure 4). Ki67 quantification in the proliferation zone revealed a comparable proliferation percentage in donor samples (64.1% ± 20%) and MINOsphere derived outhgrowths (62% ± 28%). As expected, some histologic pattern variation between different MINO lines was observed, and the variation matched patterns characteristic of each line. A sample comparison of the phenotype of derivative donor MINO line and the single cell transplant for MINO line 4 is illustrated in Figure 4. Additionally, second and third serial transplantations from the single cell MINOsphere-derived outgrowths retained their characteristic morphologies (Additional file 3).

## Discussion

**Figure 5:**
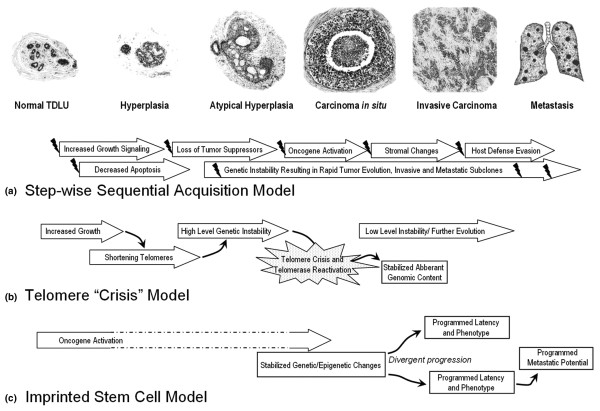
Conceptual models of breast cancer progression. **(a) **Sequential acquisition of molecular alterations with selection of advantageous 'hits' and corresponding morphologic progression (modified from Burstein and coworkers [4]). Individual 'hits' are depicted with small 'lightening bolts'. **(b) **Hyperplasia results in shortening telomeres, rapidly increasing genetic instability during 'telomere crisis', and relative stability after telomerase reactivation (modified from Chin and coworkers [15]). Individual cells reactivating telomerase and with a 'fit' genetic profile give rise to the carcinoma *in situ *(DCIS) and then the invasive carcinoma. **(c) **Genetically stable precancer stem cells are initiated via oncogene activation with divergent behavior programmed via epigenetic encoding and possible but not required genetic content changes. Intermediate morphologic and molecular events are not required for progression. These cells give rise to the DCIS and have an innate latency to invasive carcinoma and an innate metastatic potential.

The model of breast cancer progression via sequential acquisition of selectively advantageous molecular alterations with morphologically recognizable phenotypes is a convenient conceptual framework (Figure 5a). However, alternative models are possible. Progression from one lesion to the next cannot be experimentally tested in human populations, although several associations are clear. DCIS is associated with invasive cancer in close anatomic proximity. However, progression of DCIS through sequential grades and progression of invasive cancer from low to high grade or from estrogen receptor positive to negative is not common in patient pathology studies. Purported precursor lesions of hyperplasia and atypical hyperplasia are not known to carry higher risks for cancer at the site of the lesion. Instead, they are markers of risk throughout the breast. Although there is molecular evidence of clonal relationship between some of these lesions and associated cancer, the same analysis shows clonality with 'normal' mammary epithelium. Finally, the specific 'hits' that are responsible for the transition from DCIS to invasive cancer remain elusive despite extensive study.

The model proposed by Chin and colleagues [15] describes the initiation of cancer occurring through telomere shortening and genetic instability, with a pattern of genetic changes becoming stabilized in the cell that reactivates telomerase (Figure 5b). The immortalization of this cell with its unique genetic composition might be considered the 'birth' of the cancer-initiating cell [11]. Quite remarkably, their model suggests that this occurs as the tissue becomes DCIS. That is to say, the cancer stem cell [11] is 'born' at the precancer stage. Our data modify the model slightly, to show that genetic instability is not required in this process. Over-expression of a single transgene is sufficient in our genetically engineered model system. Similarly, however, the MINO model suggests that the immortalization results in the 'birth' of a precancer stem cell and that there is a stabilized programming of this cell [11]. Our data support a modified model, in which the programming stabilized in the precancer stem cell might be epigenetic as well as genetic. Also, ongoing genetic 'low level' changes and epigenetic changes may or may not contribute heterogeneity, but are not required for progression (Figure 5c).

Others have proposed that cancer with specific properties may arise in parallel rather than sequentially from the histomorphologic lesions associated with cancer risk. Mathematic modeling, supported by gene expression correlative data, demonstrates a high probability for this model in human cancer progression [16,17]. Our results are the first to document this parallel progression model experimentally. Specifically, we show that mammary precancer harbors individual cells that are capable of reconstituting the precancer tissue, and that these cells have a stable, programmed malignant potential that does not depend on sequential acquisition of genetic alterations. Each of the precancer lines is derived from genetically identical mammary tissue but develop divergently. The divergent lines, derived from biopsy transplantation, harbor unique and consistent malignant transition latencies and metastatic capacity. These findings confirm a precancer 'stem' cell, as defined by the AACR working group [11], as the origin of invasive cancer. Furthermore, the data challenge the concept of sequential acquisition of selectively advantageous traits through genetic 'hits'. Instead, the data support a divergent evolution of precancer stem cells with intrinsic programmed latency to invasion and metastatic potential.

Because human breast cancer progression has been associated with genetic instability, we initially hypothesized that we would find genetic changes related to precancer-to-carcinoma progression in the MINOs, either common to several of the lines or consistently found within a single line. This would also correlate with data from another mouse model of DCIS, p53-null mammary epithelial transplant, which exhibits genetic instability and heterogeneous progression to a spectrum of carcinomas [18,19]. Instead, in our model, we found no consistent CGH changes in the transition from precancer mammary intraepithelial neoplasia to invasive carcinoma. In fact, most invasive carcinomas were nearly identical in their genomic content to the paired MIN precancer [10] (and Additional file 1). Specifically, several of the lines exhibited a normal CGH profile, suggesting a euploid and unaltered genomic content. In others, there were few changes and most were whole chromosome gains. In addition, CGH analyses detected differences between the six independently derived and maintained lines of MINO. Changes seen in each line were consistent between separate animals within a transplant generation and from one generation to the next, with up to three serial transplant generations studied. Furthermore, there are no changes in common between lines or related to progression from MINO to tumor. Therefore, the genomic changes are perhaps best regarded as markers of clonal cell populations rather than mechanistically important genetic changes. Taken together, these CGH profiles indicate that the individual MINO lines harbor stable genomic changes consistent with a common origin (clonality).

The 'telomere crisis' hypothesis [15,20] is supported, in part, by our studies. Despite the absence of high levels of genetic instability in our model system, telomerase activity and restabilizaton at the inception of the precancer is confirmed. The differences between telomerase activity in our MINO model and human tissue may be related in part to species differences. In fact, mouse cells have longer telomeres than human cells, and are less susceptible to genetic instability caused by telomere shortening [21]. Nevertheless, normal mouse mammary tissue will senesce after serial passage [22], and this is probably related to telomere shortening [23]. Hyperplastic lesions in the mouse mammary gland that do not senesce after many serial passages have been identified. Some of these lesions never progress to invasive carcinoma, but others do progress, and this defines them as 'precancers' [8]. Because immortalized hyperplasia as well as true precancers may have stabilized telomeres, this property is not sufficient for cancer initiation. In the MINO model, the initiating stimulus is provided by the transgene PyVmT (polyoma virus middle T). Interestingly, however, PyVmT alone is not sufficient for immediate transformation to cancer. The intermediate MIN outgrowths express PyVmT at levels equal to the carcinomas [7]. So, the combination of PyVmT expression and telomere stabilization is sufficient for establishment of the precancer, but transformation to cancer requires additional factors that are preprogrammed at the point of precancer initiation, and not spontaneous 'hits' in a susceptible tissue/cell.

A great deal of interest has recently been focused on identifying the cancer 'stem' cell [11] in a wide variety of cancers [24]. Attention has emphasized markers identifying the cells with self-renewal and tumor-reinitiating capacities. These studies require proof of capacity using a functional assay, usually tumor growth in xenograft transplants. Here, we present a refined functional assay similar to findings reported for normal mouse mammary stem cells by using cleared mammary fat pad transplantation [25]. Normal, hyperplastic, and precancerous mammary tissues grow only in this context, whereas cancers will also grow in ectopic sites [7]. The use of immune intact syngeneic recipient mice also contributes to a more realistic model of cancer progression [13]. The refined functional assay and the use of a highly characterized, well validated mouse model of cancer progression permits a precise assessment of the earliest origins of these interesting cells.

The importance of the stroma or cell niche is well documented in epithelial neoplasia [26,27]. In this study we have utilized two highly specialized stromal environments in the functional analysis of cell potential: the cleared mammary fat pad and Matrigel in organoid cell culture. The addition of Matrigel in the non-adherent culture was adopted primarily for technical reasons to ensure that individual cells were prevented from contacting one another and to facilitate transplantation of single cell derived organoids. Matrigel is well known to have specific growth and differentiation inducing effects, however, and may have had specific effects on susceptible cells in our dissociations. Although we have transplanted MINO cells directly into the cleared fat pad many times, they have always been transplanted as tissue biopsies [7,8,10,14]. Some of the array of cell types transplanted in this way may contribute to conditioning the stroma for growth of the reinitiating cells, and Matrigel may be providing a similar condition. Furthermore, the experimental stochiometery may result in an underestimate of the rate of initiating cell potential. Regardless of the requirement for specific stroma conditions, these data prove that a single cell can reinitiate the precancer and maintain the programmed transformation to cancer.

## Conclusion

The experiments described here demonstrate that the precancerous MINO is the product of a genetically stable precancer initiating cell. These experiments are consistent with the hypothesis that the full biological potential of cancer is pre-encoded by the time that a microscopic precancer (DCIS) is found. It suggests that subsequent events involve epigenetic phenomena and that gross genomic change may play a role in programming but is not necessary for neoplastic progression to malignancy and metastasis. Our model suggests that the risk for advancing to invasive breast cancer from human DCIS will be predictable at the precancer stage, perhaps requiring an understanding of the interactions between the DCIS epithelium and stroma.

## Abbreviations

bp = base pairs; CGH = comparative genomic hybridization; CK = cytokeratin; DCIS = ductal carcinoma *in situ*; DMEM = Dulbecco's modified Eagle's medium; MIN = mammary intraepithelial neoplasia; MINO = mammary intraepithelial neoplasia outgrowth; PCR = polymerase chain reaction; SMA = smooth muscle actin; TRAP = telomerase repeat amplification protocol.

## Competing interests

The authors declare that they have no competing interests.

## Authors' contributions

PD performed all cell dissociation and culture experiments, performed immunohistochemistry, analyzed and interpreted data, and drafted portions of manuscript. JGH performed initial CGH experiments, assisted with genetic interpretations, and reviewed the manuscript. JQC performed the TRAP assay, managed mouse colonies and tissue archives, and assisted with cell culture. LJTY assisted with transplantations and evaluation of *in vivo *phenotypes. RDC assisted with histopathology and immunohistochemistry analyses, consulted on all data interpretation, advised regarding experimental design, and conoducted critical editing of the manuscript. ADB was responsible for primary experimental design, coordination of experiments, interpretation of all histology/immunohistochemistry, and – with PD – drafted the manuscript and prepared figures.

## Supplementary Material

Additional file 1 A tif file summarizing CGH and microsatellite analysis by line. For each line, samples of MINO and the matched tumor were analyzed by BAC array CGH and multiplexed PCR for microsatellite changes. For CGH results, changes that were seen in all of the MINO or tumor samples from each line are depicted in solid color, and changes seen in less than 100% of samples in cross-hatched color with the fraction and total number given below. Whole chromosome gains (WCGs) are shown as rectangular boxes, whereas smaller amplifications are depicted as ovals with (+) symbols and deletions as ovals with (-) symbols. The approximate relative chromosomal location is mapped with the chromosomes depicted along the left edge. The microsatellites are depicted as small boxes, with two at each position depicting the two alleles. Empty squares are microsatellites matching the normal control sample (wild-type FVB/n) and filled squares are changes in microsatellite length from normal. In most cases, the length change was seen in one of the two alleles, but in a few areas both alleles were altered as in MINO line 8wD at centromeric chromosome 2 (top of chromosome 2 as depicted) where both squares are filled. **Line 4w6 was not studied by microsatellite analysis. Click here for file

Additional file 2 An Excel file showing a microsatellite analysis of MINO and matched tumor. Listed are all microsatellite PCR nucleotide length results. Open cells indicate failed or unreadable reactions. Click here for file

Additional file 3 A tif file showing serial transplantation of MINOSphere-derived outgrowth. Histology (hematoxylin and eosin stained, formalin-fixed, paraffin-embedded 4 μm sections) of donor (A, D), second (B, E), and third (C, F) serial transplantations from the single cell MINOsphere derived outgrowths from MINO line D (panels A, B, and C) and MINO line 4 (panels D, E, and F). Line D (panels A, B, and C) all show a microacinar pattern and transplant attempts resulted in growth of 75% (*n *= 8) of second serial and 100% (*n *= 16) of third serial transplant attempts. MINO line 4 (panels d, e, and f) shows a solid lobulated pattern typical of line 4 and transplant attempts resulted in growth of 67% (*n *= 6) and 100% (*n *= 8) of third serial transplants. All panels are identical magnification, with the 50 μm scale bar shown (panel f). Click here for file

Additional file 4 A Word document outlining the CGH procedure. Click here for file
