# Supplementary figures and images for: Mammary carcinoma behavior is programmed in the precancer stem cell
Source: Breast Cancer Res. 2008 Jun 3;10(3):R50. doi: 10.1186/bcr2104 (PMC2481504; doi:10.1186/bcr2104)

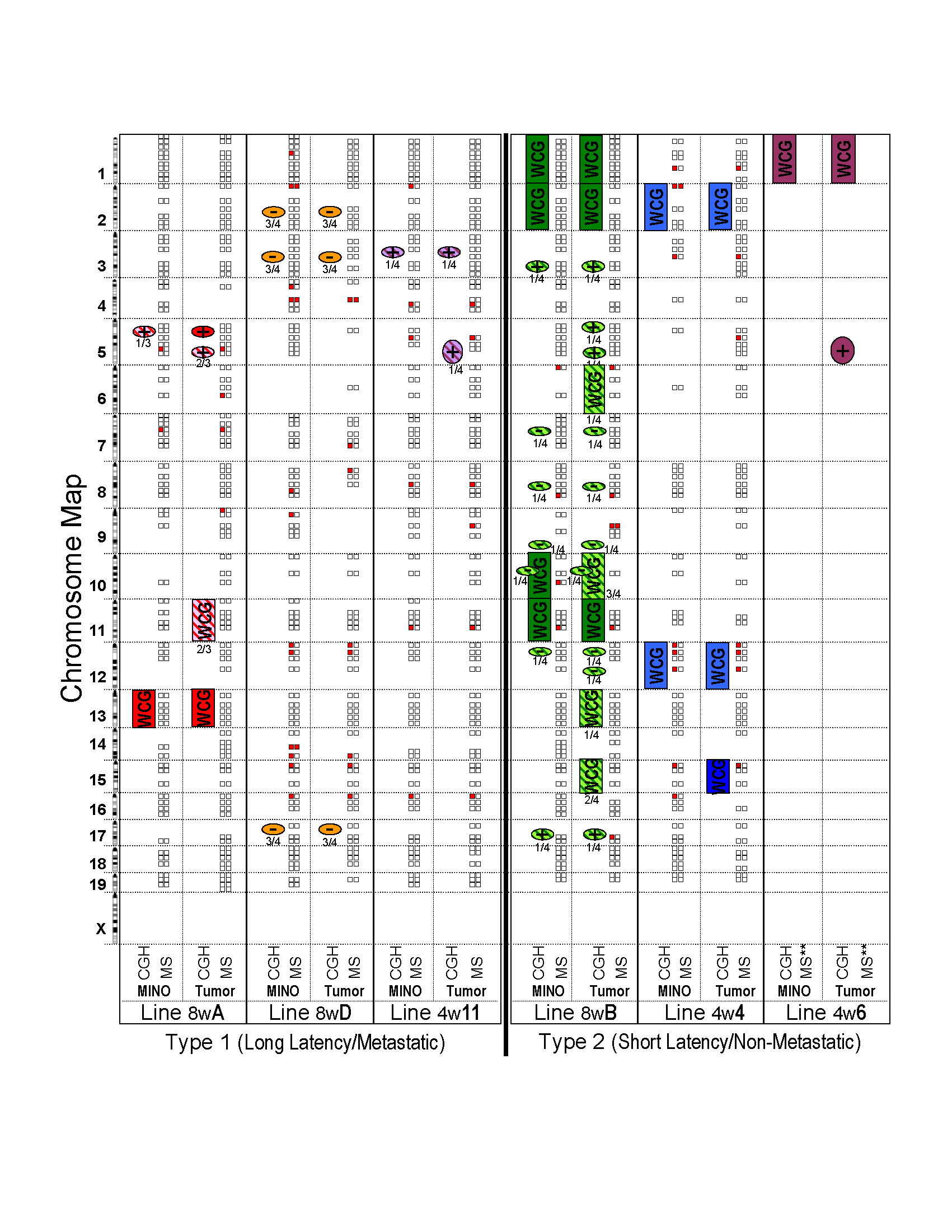

Supplement: Additional file 1 — A tif file summarizing CGH and microsatellite analysis by line. For each line, samples of MINO and the matched tumor were analyzed by BAC array CGH and multiplexed PCR for microsatellite changes. For CGH results, changes that were seen in all of the MINO or tumor samples from each line are depicted in solid color, and changes seen in less than 100% of samples in cross-hatched color with the fraction and total number given below. Whole chromosome gains (WCGs) are shown as rectangular boxes, whereas smaller amplifications are depicted as ovals with (+) symbols and deletions as ovals with (-) symbols. The approximate relative chromosomal location is mapped with the chromosomes depicted along the left edge. The microsatellites are depicted as small boxes, with two at each position depicting the two alleles. Empty squares are microsatellites matching the normal control sample (wild-type FVB/n) and filled squares are changes in microsatellite length from normal. In most cases, the length change was seen in one of the two alleles, but in a few areas both alleles were altered as in MINO line 8wD at centromeric chromosome 2 (top of chromosome 2 as depicted) where both squares are filled. **Line 4w6 was not studied by microsatellite analysis. [file bcr2104-S1.tiff]

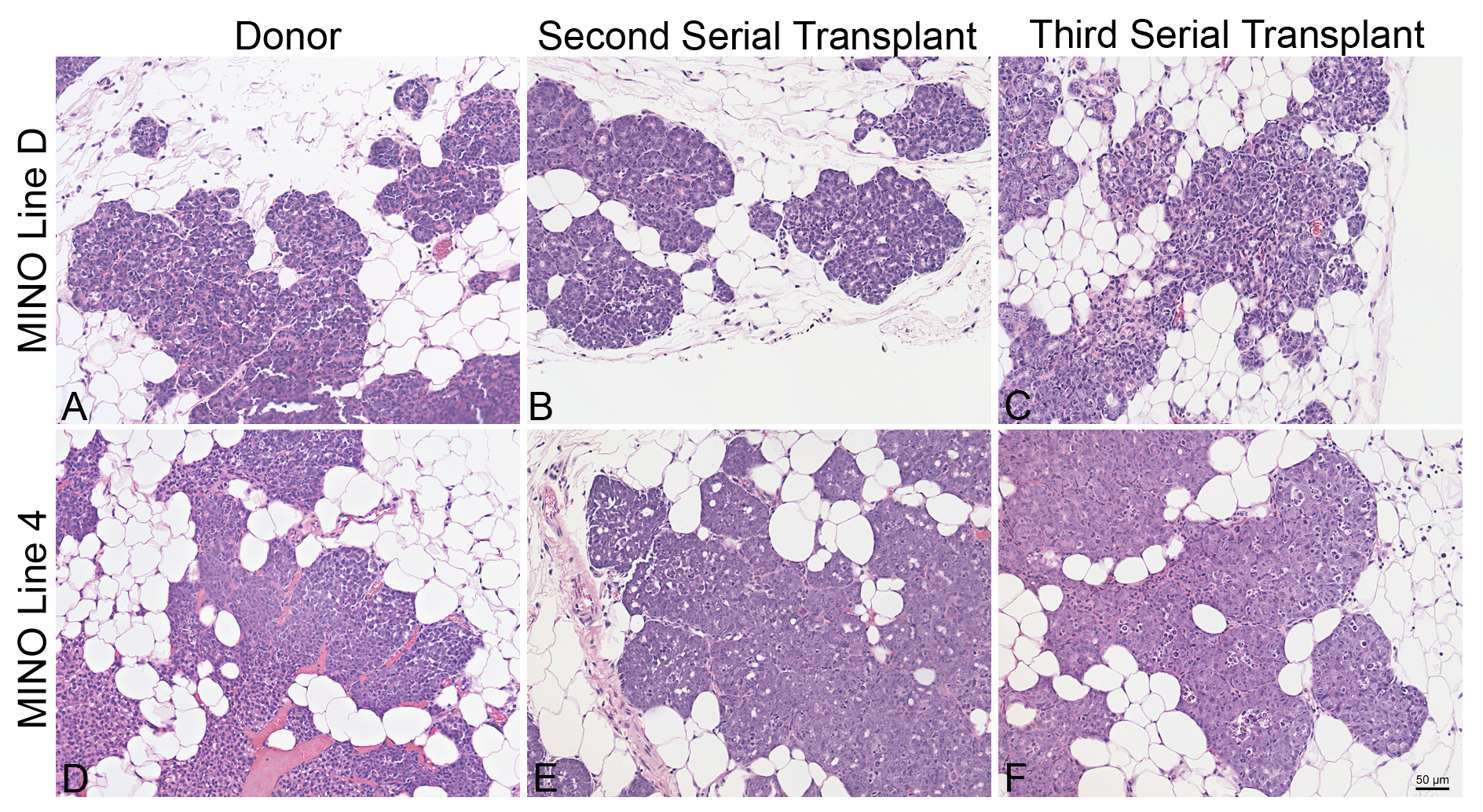

Supplement: Additional file 3 — A tif file showing serial transplantation of MINOSphere-derived outgrowth. Histology (hematoxylin and eosin stained, formalin-fixed, paraffin-embedded 4 μm sections) of donor (A, D), second (B, E), and third (C, F) serial transplantations from the single cell MINOsphere derived outgrowths from MINO line D (panels A, B, and C) and MINO line 4 (panels D, E, and F). Line D (panels A, B, and C) all show a microacinar pattern and transplant attempts resulted in growth of 75% (n = 8) of second serial and 100% (n = 16) of third serial transplant attempts. MINO line 4 (panels d, e, and f) shows a solid lobulated pattern typical of line 4 and transplant attempts resulted in growth of 67% (n = 6) and 100% (n = 8) of third serial transplants. All panels are identical magnification, with the 50 μm scale bar shown (panel f). [file bcr2104-S3.tiff]
