# Supplementary material for: Mammary carcinoma behavior is programmed in the precancer stem cell
Source: Breast Cancer Res. 2008 Jun 3;10(3):R50. doi: 10.1186/bcr2104 (PMC2481504; doi:10.1186/bcr2104)
Supplement: Additional file 4 — A Word document outlining the CGH procedure. [file bcr2104-S4.doc]

**Comparative genomic hybridization**

1 µg of DpnII-digested tumor DNA and reference DNA (female FVB) was labeled with Cy3 or Cy5, respectively (Amersham, NJ, USA) using the Bioprime random-primed labeling kit (Invitrogen, CA, USA). Unincorporated nucleotides were removed using Sephadex G-50 columns (Amersham, NJ, USA), and tumor and reference samples were combined, precipitated with 50 µg of mouse Cot-1 DNA, and resuspended in 60 µl of hybridization solution (10% dextran sulfate, 50% formamide, 2 x SSC, 4% SDS, and 10 µg/µl yeast tRNA). Preannealed DNA samples were then applied to arrays fitted with 2.4 cm2 EasiSeal gaskets (Continental Lab Products, San Diego, CA). Slides were placed in individual, prewarmed hybridization chambers (plastic slide holders) containing Whatmann paper soaked with 750 µl of 2 x SSC, 0.1% SDS to prevent evaporation and hybridized for a minimum of 36 h at 37°C on a slowly rocking platform. After hybridization, slides were washed, counterstained with 4',6-diamidino-2-phenylindole, and imaged using a custom-built CCD camera. TIFF images were segmented, and spot statistics were calculated using custom software.
